# Beyond Streamflow: Call for a National Data Repository of Streamflow Presence for Streams and Rivers in the United States

**DOI:** 10.3390/w13121627

**Published:** 2021-06-09

**Authors:** Kristin L. Jaeger, Konrad C. Hafen, Jason B. Dunham, Ken M. Fritz, Stephanie K. Kampf, Theodore B. Barnhart, Kendra E. Kaiser, Roy Sando, Sherri L. Johnson, Ryan R. McShane, Sarah B. Dunn

**Affiliations:** 1 U.S. Geological Survey, Washington Water Science Center, Tacoma, WA 98402, USA; 2 U.S. Geological Survey, Idaho Water Science Center, Boise, ID 83702, USA; 3 U.S Geological Survey, Forest and Rangeland Ecosystem Science Center, Corvallis, OR 97331, USA; 4 Office of Research and Development, U.S. Environmental Protection Agency, Cincinnati, OH 45268, USA; 5 Department of Ecosystem Science and Sustainability, Colorado State University, Fort Collins, CO 80526, USA; 6 U.S. Geological Survey, Wyoming-Montana Water Science Center, Helena, MT 59601, USA; 7 Department of Geosciences, Boise State University, Boise, ID 83725, USA; 8 U.S. Forest Service, Pacific Northwest Research Station, Corvallis, OR 97331, USA

**Keywords:** perennial, non-perennial, intermittent, ephemeral, surface flow, headwaters, crowdsourcing, database

## Abstract

Observations of the presence or absence of surface water in streams are useful for characterizing streamflow permanence, which includes the frequency, duration, and spatial extent of surface flow in streams and rivers. Such data are particularly valuable for headwater streams, which comprise the vast majority of channel length in stream networks, are often non-perennial, and are frequently the most data deficient. Datasets of surface water presence exist across multiple data collection groups in the United States but are not well aligned for easy integration. Given the value of these data, a unified approach for organizing information on surface water presence and absence collected by diverse surveys would facilitate more effective and broad application of these data and address the gap in streamflow data in headwaters. In this paper, we highlight the numerous existing datasets on surface water presence in headwater streams, including recently developed crowdsourcing approaches. We identify the challenges of integrating multiple surface water presence/absence datasets that include differences in the definitions and categories of streamflow status, data collection method, spatial and temporal resolution, and accuracy of geographic location. Finally, we provide a list of critical and useful components that could be used to integrate different streamflow permanence datasets.

## 1. Introduction

In the past 30 years, managing the flow regime of rivers has emerged as a primary focus for protecting species, sustaining ecosystem processes, and satisfying the consumptive demands of human society [1–3]. More recently, attention has turned toward non-perennial streams—streams that periodically have no surface flow [4–6]—and the importance of quantifying when and where streams do or do not contain surface flow (i.e., streamflow permanence; [5,7–9]). Non-perennial streams account for the majority of channel length across networks at a global scale [5,10,11] and are predominantly represented by headwaters (2nd order or smaller). In spite of their prevalence, non-perennial streams and headwater streams in general are vastly underrepresented in existing stream gaging networks in the United States [12–15].

**Figure 1.**
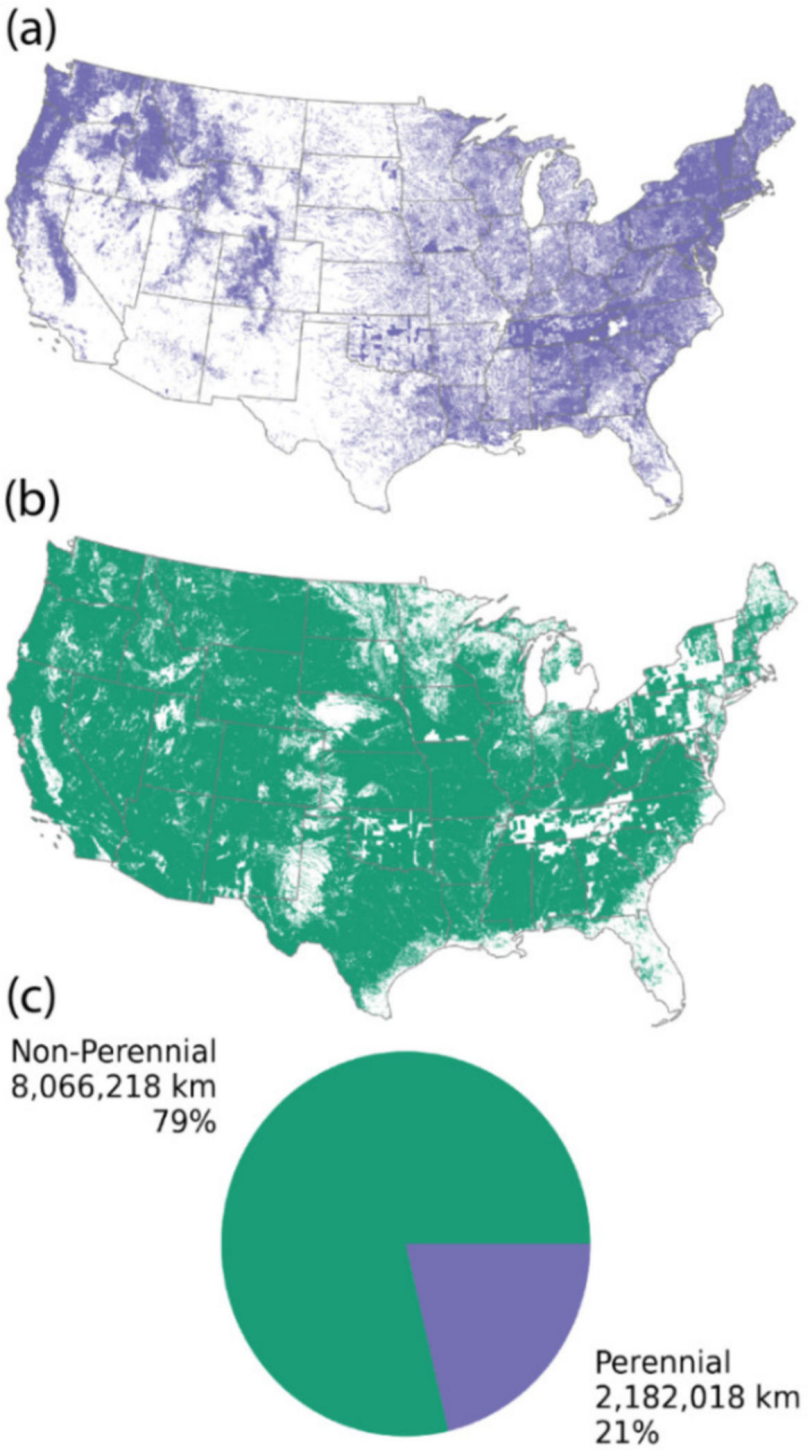
Maps showing the distribution of perennial (**a**) and non-perennial (**b**) streams in the Continuous United States (CONUS) from the U.S. Geological Survey National Hydrography Dataset (NHD) High-Resolution Hydrography [32] and the total and relative lengths of each category (**c**).

Although most stream gages are equipped to monitor stream discharge, here, we focused on streamflow permanence and how to better quantify and assemble information that can inform our understanding of streamflow permanence outside of traditional stream gaging networks [14]. Streamflow permanence can be characterized by the frequency and duration of surface flow in streams and includes both a spatial and temporal component [16]. Understanding the streamflow permanence of headwater streams is important for regulatory determinations and policy [17–19], water resource management [20], and ecological processes [4,10,21–23]; however, streamflow data remain sparse for headwater non-perennial streams, and current streamflow permanence classifications (perennial or non-perennial) often disagree with in situ observations [24–26]. Given the abundance of non-perennial streams both within the U.S. (Figure 1) and globally [11], and the prospect of declining stream flows in the future [16,27,28], accurately characterizing streamflow permanence represents a major challenge to understanding water availability now and throughout the 21st century [24,25]. Consequently, there have been several recent calls to fill this critical data gap [29–31], with van Meerveld et al. [31] aptly likening the lack of knowledge of non-perennial headwater streams to, “a puzzle with most pieces still under the carpet.”

The National Water Information System, (NWIS, waterdata.usgs.gov accessed on 15 February 2021) is a high-quality, consistent database that reports discharge from the stream gaging network of the U.S. Geological Survey (USGS) and is the foundation for much of what is known about streamflow regimes in the U.S. However, because of the bias toward perennial and larger rivers [13], the existing USGS stream gaging network is insufficient to accurately capture the spatial extent of streamflow permanence. Expanding the stream gaging network using non-traditional gaging techniques that include simpler measurement approaches can be useful to increasing our understanding of streamflow permanence in headwater streams. For example, France developed the Observatoire National des Etiages (ONDE) network to address the data gap in headwaters. It complements the existing national stream gaging network HYDRO database, but focuses on discrete, repeat measurements of hydrological state for 3350 tributary streams [31]. Programs like these are costly and require substantial investment of resources. However, in the absence of a national monitoring effort in the U.S. that targets headwaters and non-perennial streams, a combination of existing, but diverse, datasets and recently developed mobile applications for the collection of surface flow conditions offer a promising, low-cost opportunity to fill this streamflow data gap in headwaters and non-perennial streams. Accordingly, the stage is set to integrate diverse and growing datasets into a single, consistent database that is readily available to the general public, managers, and scientists.

The objective of this paper is to make the case for such a database founded on simple, categorical field observations of the presence or absence of surface water for improved characterization of streamflow permanence regimes of non-perennial systems. To address this objective, the following are highlighted: (1) information on surface water presence that may be gained from existing surveys that were not originally intended to provide such information; (2) new information from emerging collection protocols and mobile applications intended specifically for this purpose; and (3) the challenges that lie ahead for data integration and database development of surface water presence/absence observations that can advance our understanding of streamflow permanence regimes of headwater streams. We restricted our application to the continuous United States with a call for a national data repository, but recognize the need for a universal data repository that spans the globe given the worldwide prevalence of non-perennial streams, and apparent scarcity of streamflow data for these non-perennial streams [11].

## 2. Background

**Figure 2.**
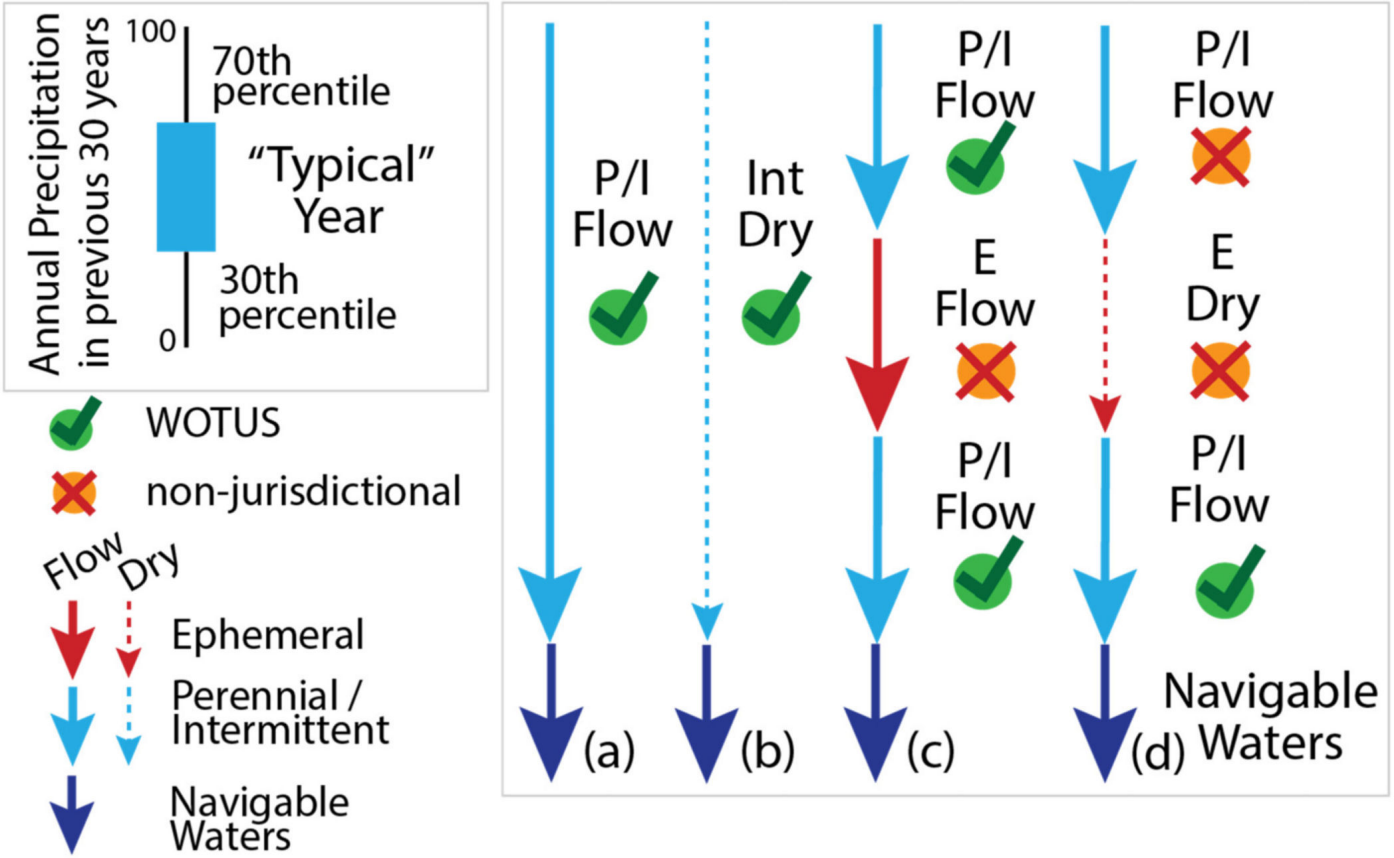
Perennial streams (P) are assumed to always have flow in a typical year and are considered WOTUS (Waters of the United States) if they contribute surface water to navigable waters (**a**). Typical flow is defined as having precipitation that is within the 30th and 70th percentile based on the previous 30 years. Intermittent streams (I) are considered WOTUS if they contribute surface water to navigable waters in a typical year regardless of whether they are flowing (**a**) or dry (**b**). Ephemeral streams (E) are never considered WOTUS (**c**,**d**). If a perennial or intermittent stream (P/I) is flowing into an ephemeral stream that flows during a typical year, then the perennial or intermittent stream is considered WOTUS (**c**), whereas if a perennial or intermittent (P/I) stream is flowing into an ephemeral stream that is always dry in a typical year, that upstream stream is not considered WOTUS (**d**).

Determining streamflow permanence has important implications for water resources including water availability, water quality, and ecosystem processes [4,20,21]. Characterizing streamflow permanence including identifying where, when, and for how long streams go dry, therefore, has important consequences for numerous regulatory contexts, for example, the U.S. federal level protection of waterways under the Clean Water Act (i.e., Waters of the United States or WOTUS). Since 2006, the legal status of waterways considered federally protected has in large part hinged on the duration and frequency of surface flow in reaches connecting tributaries to navigable water bodies [17–19]. As of June 2020, the federal policy defining WOTUS (“Navigable Waters Protection Rule,” [9]) considers jurisdictional tributaries as those that are perennial or intermittent (Table 1) and contribute surface water flow to a traditional navigable water or territorial sea in a typical year (Figure 2). For regulatory purposes, a typical year is based on the 30th to 70th percentile of climate data such as total annual precipitation over the last 30 years (i.e., rolling 30-y record). The rule excludes ephemeral streams (i.e., flow only in direct response to precipitation, Table 1) and breaks (e.g., culverts, pipes, dams, underground streams, boulder fields) from WOTUS. The rule includes upstream perennial and intermittent streams that have connections to downstream WOTUS through ephemeral streams or breaks, but only if the connecting ephemeral stream carries flow in a typical year (Figure 2c), otherwise the upstream perennial and intermittent streams are excluded from WOTUS (Figure 2d, [8]). Breaks such as buried streams or caves, by definition, do not carry surface flow, but must carry flow to a downstream WOTUS for the upstream perennial or intermittent streams to be considered jurisdictional. The implications of the 2020 rule and importance of appropriately mapping streamflow permanence has been examined elsewhere [26,33]. Here, we highlight that the particular details on surface flow timing and spatial configuration of flowing and nonflowing stream segments relative to each other (e.g., Figure 2c,d) further underscores the need for tools that include accurate mapping and modeling of surface flow presence/absence to implement policies and management decisions (e.g., jurisdictional determinations, water quality standards, riparian buffer rules) based on surface flow regime and network position.

In the U.S., the first delineations of streamflow permanence, most of which are still used today [25], resulted from ground-based surveys and hand-drawn maps of streams produced by the USGS. These hand-drawn maps were digitized to create the National Hydrography Dataset (NHD), which maps the locations of streams and rivers. Streamflow permanence classifications (hydrographic category; Table 1) are attributed to these flowlines. The definitions of these classes have changed through time, creating subsequent inconsistencies between classifications (reviewed by [25]; Table 1). Current definitions focus on the presence or absence of surface water instead of surface flow and formally include a definition for ephemeral streams.

Given the well-known issues with hydrography and streamflow permanence classifications within the NHD [15,24,25], the USGS has established a NHD Stewardship Program in partnership with federal, state, and local agencies that leverages local knowledge of watersheds to facilitate ongoing updates that include stream and river flowline attributes including streamflow permanence classifications [38]. However, the task to update the hydrography and streamflow permanence classifications substantially outsizes the capacity and resources of stewardship programs so that for at least streamflow permanence classifications, classifications are sometimes decades out of date [25]. The dynamic nature of streamflow permanence in time and space in headwater streams [39–42] suggests that information on location, extent, and timing of streamflow permanence as well as changes in permanence through seasons and across years are needed.

There are a variety of ways in which information on permanence can be recorded and updated. In some cases, patterns of streamflow permanence can be determined using instrumental records or remotely-sensed imagery and Light Detection and Ranging (LiDAR) [43–45]. Direct ground-based observations can validate these approaches [46] and are arguably the most reliable way to determine streamflow conditions, particularly for smaller streams and in areas of dense canopy cover. Dense cover is common in non-perennial streams where vegetation height may easily be greater than channel width.

In the absence of a stream gaging network, direct, field-based observations of surface water presence can be collected by two approaches. The first approach leverages data from existing stream surveys conducted for other purposes (e.g., stream habitat surveys, timber sales; [47]). The second approach uses several new tools available for engaging the general public (CrowdWater [48], Stream Tracker [29]), and agency personnel and other scientists (FLOwPER [49]) in collecting data on when and where streams have surface flow (Table 2). Collectively, these data sources could be combined to increase the geographic and temporal resolution of hydrologic condition in headwater streams for developing streamflow duration assessment methods [50], expanding the understanding of controls on streamflow permanence [16], and contributing to updating information used for land and water management decisions. This information is critical for land use zoning, conserving imperiled species, implementing water quality standards, and determining stream buffer requirements in preparation for forest harvest or other land and water resource actions [19,51].

## 3. Surveys That Provide Incidental Information on Streamflow Permanence

**Figure 3.**
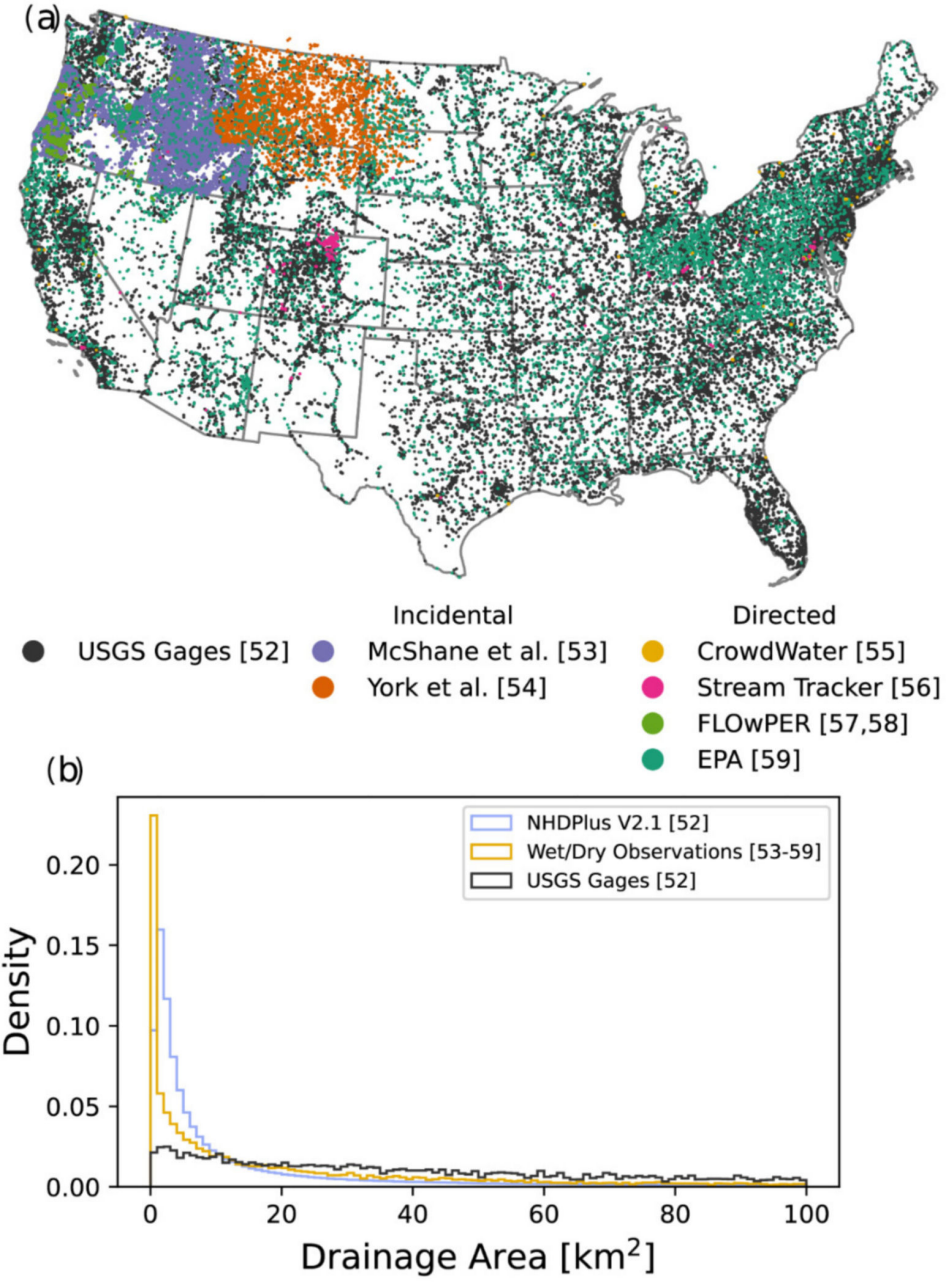
Map (**a**) of USGS NHDPlus V2.1 stream gaging network in CONUS [52] and wet/dry observations aggregated from stream survey incidental information [53,54], EPA probabilistic stream surveys, dedicated streamflow permanence surveys via mobile applications CrowdWater [55], Stream Tracker [56], and FLOwPER [57,58], and EPA probabilistic stream surveys (NRSA 2008–2009 and 2013–2014, WSA-Western EMAP 2000–2004, EMAP Mid-Atlantic Highlands Assessment 1993–1996, Mid-Atlantic Integrated Assessment 1997–1998, Kansas Regional EMAP 2001, and Eastern Cornbelt Regional EMAP 1995) [59], and density plots (**b**) of wet/dry observations, gages, and NHDPlus V2.1 flowlines in CONUS [52] drainage areas less than 100 km^2^.

Documenting surface water presence is often a standard component of stream surveys that are conducted for a range of basic science or applied management objectives (Table 2). However, incidental information on streamflow status that includes surface water presence or location of headwater streams is typically not compiled into a central database and instead resides within fragmented institutional or agency databases on local computers or in paper archives such as field notes. Furthermore, information from these surveys may not be part of the NHD Stewardship program. Consequently, databases are often siloed within the host agency and can remain either unknown or not readily available for access outside the agency, even though they may be the only streamflow information for this part of the stream network (Figure 3).

One example of a large data collection effort that has resulted in incidental streamflow permanence data is the Idaho Department of Environmental Quality Beneficial Use Reconnaissance Program (BURP; https://www.deq.idaho.gov/water-quality/surface-water/monitoring-and-assessment/ (accessed on 4 June 2021)). BURP was initiated in 1993 to evaluate the condition and uses of Idaho water bodies to support the Clean Water Act. Every summer, data on ecological conditions and water quality are collected in streams across the state. A no-flow status is recorded for sites that do not have surface flow during the summer survey. Similarly, a host of broad-scale stream monitoring programs supported by federal land management agencies are not necessarily focused on streamflow permanence, but do provide information on sites with no surface flow (e.g., monitoringresources.org (accessed on 4 June 2021)). Other examples include local field surveys associated with timber sales on federal, state, and private forest land that require field verification on the location of streams and their streamflow status for riparian management zone determinations. Data collection is often in rugged, hard-to-access terrain, making these data all the more valuable, as no information would be collected in these locations otherwise.

## 4. Surveys Designed Specifically for Identifying Surface Water Presence and Characterizing Streamflow Permanence

In response to a general lack of data about streamflow permanence, some research groups have recently developed survey methods that allow the general public or agency personnel to collect hydrological observations in a consistent and standardized manner. These efforts provide an opportunity for rapid data collection over potentially extensive geographic regions as a low-cost approach toward filling this data gap to characterize streamflow permanence (Figure 3). Dedicated surveys include conventional field data collection approaches to characterize streamflow permanence [50], whereas other efforts leverage crowd sourcing approaches with readily available mobile device applications to collect rapid, visual observations of surface water presence [29].

One example of a dedicated field survey is Streamflow Duration Assessment Methods (SDAMs), which are rapid, stream-scale indices or models that use physical and/or biological indicators to predict flow duration class [50]. Such models are developed from study sites with either continuous or periodic observations of surface flow presence/absence [60,61].

**Figure 4.**
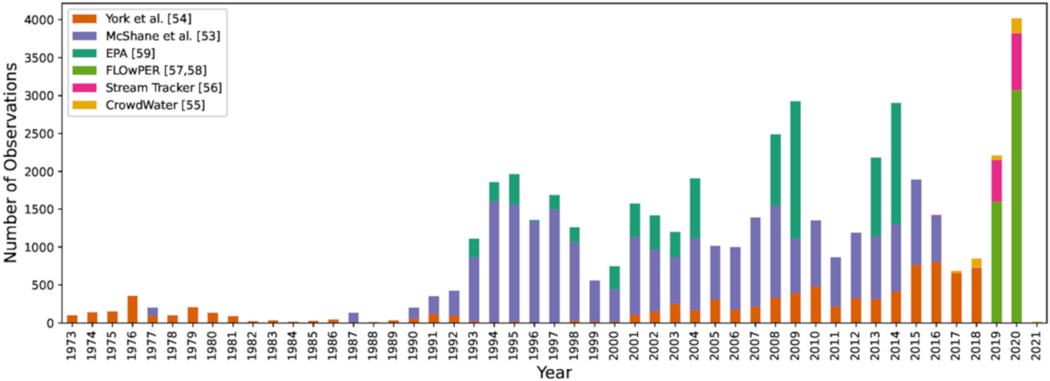
Time series of surface water presence observations colored by source. Note, only CrowdWater observations located in the United States and shown in Figure 3 are included here. McShane et al. [53] and York et al. [54] are flow/no flow observation datasets originally aggregated from several local, state, tribal, and federal agencies to support PROSPER model development.

Stream Tracker, CrowdWater, and FLOwPER are surveys that specifically focus on crowdsourced, streamflow presence observations collected using mobile device applications. Crowdsourcing can be an effective mechanism for rapidly generating sizable datasets on streamflow permanence that rival or exceed existing datasets derived from other agency surveys (Figure 4). Stream Tracker is open to any interested person, who can upload observations on streamflow conditions (flow, no flow, standing water) using either a website or mobile phone app. As of January 2021, the Stream Tracker database includes over 6000 observations across nearly 1000 locations, mostly in the U.S. [56]. The observations are housed on both CitSci.org (accessed on 15 January 2021) and Anecdata.org (accessed on 15 January 2021), which are both open access databases for community science. The data have been used by agencies to identify monitoring locations for studies on streamflow permanence and to identify potential locations for native fish reintroduction. CrowdWater [55] is another mobile application that facilitates a range of hydrological observations that include water level, soil moisture, plastic pollution, and streamflow estimation in addition to flow/no flow observations. As of January 2021, the CrowdWater database has collected a total of 18,900 observations, of which approximately 7400 are for surface water presence observations and include observations on all continents [55].

Finally, FLOwPER was developed in a collaborative project between USGS, USFS Research and Development (USFS R&D), and the Bureau of Land Management (BLM) as a customized field form in a mobile application for a target audience of agency field personnel who are already conducting field surveys in or near streams [49]. FLOwPER observations (continuous flow, discontinuous flow, dry) are uploaded into the FLOwPER Database within the ArcGIS Online environment (AGOL), which are immediately available to all FLOwPER contributors. Additionally, the database is publicly available in USGS ScienceBase through approximately annual updates to the FLOwPER Database [57]. As of January 2021, the FLOwPER database includes over 5000 observations at more than 4000 locations in the Pacific Northwest [57]. These data are currently being used for the development of empirical streamflow permanence models.

## 5. Applications of Streamflow Presence Data

Stream survey datasets that reside in various forms of electronic and paper records have a broad range of potential uses that expand beyond the original agency goals (Table 3). For example, these observations can be used to develop streamflow permanence models [7,62] that can be applied to water availability and ecological function applications (Table 3). Historical reports and field observations by USFS field personnel were the foundation of a streamflow permanence prediction model for a mountain catchment in Montana [62]. Expanding on this approach, approximately 24,300 flow/no flow observations in the Pacific Northwest Region were compiled from 11 disparate agency datasets [53]. A subset of these observations were used for the development of the streamflow permanence model, PROSPER, which provides spatially continuous probabilities of year round flow for streams aligned with the NHD medium resolution stream grids for the Pacific Northwest Region [7]. This dataset has also been used to evaluate the accuracy of existing streamflow permanence classifications [11,25].

Expansion of the PROSPER model to the Upper Missouri River Basin resulted in another data aggregation effort, which included data from the Water Quality Portal (https://www.waterqualitydata.us/ (accessed on 4 June 2021)). The Water Quality portal is a cooperative service sponsored by USGS, USEPA, and NWQMC (National Water Quality Monitoring Council) and provides access to individual datasets related to water quality, many of which include information on streamflow presence/absence. However, these datasets were generated with different objectives, methods, and are not readily integrated. Nevertheless, approximately 35,800 streamflow status data points were extracted from the Water Quality Portal and processed [54], a subset of which was used for the PROSPER model development. This approach to aggregate disparate datasets of flow/no flow observations across agencies, organizations, and academic institutions is similar to the dedicated data aggregation effort of crowdsourced stream water temperature that is the basis for the NORWeST model for the western United States [63].

Other underutilized datasets for streamflow permanence mapping are state and EPA regional and national probabilistic surveys (Environmental Monitoring and Assessment Program [EMAP], National Wadable Stream Assessment [NWSA], and the National Rivers and Stream Assessment [NRSA]) to characterize the condition of rivers and streams in the United States [64–66]. These surveys were intended to characterize the condition of perennial rivers and streams during the summer. The assessment streams were geographically stratified and probabilistically sampled based on the 1:100,000-scale NHDPlus stream segments. When field crews found that ≤50% of the stream length of a reach had surface water, sampling followed a modified protocol for interrupted streams [67]. Compiling information from these datasets regarding which streams did and did not have surface water would improve flow permanence mapping across the U.S. One-time observations spatially distributed throughout an area of interest can be used to provide an annual probability of year round flow (e.g., [7,62]), while repeat observations throughout a season of interest can be used for predictions on the timing or duration of no flow conditions.

Surface water presence observations have useful applicability to existing hydrologic modeling as an alternative means of model calibration and validation (e.g., [68,69]). Empirical data (e.g., surface water presence observations) can be combined with theoretical hydrologic models to create more accurate hybrid models for predicting streamflow regimes and watershed storage. For example, Williamson et al. [68] used flow-state sensors to identify flow/no flow periods to calibrate the simulated saturation deficit in a spatial hydrologic model (TOPMODEL). Sufficient spatial and temporal density of surface water presence could present opportunities to estimate streamflow permanence with other process-based models (e.g., DHSVM, RHESSys, ParFlow). More streamflow presence observations would allow for predictive streamflow permanence estimates to be generated at fine spatial scales using appropriate modeling methods.

Finally, with the advent of remote sensing data products at increasingly higher temporal and spatial resolution (e.g., Sentinel-2 [70], Planet [71]), observations of surface water presence can provide critical verification data, collectively creating the opportunity to substantially expand the geographical extent for estimating surface water conditions. Remote sensing has proved useful for the identification and inventory of water features and conditions [72]. However, field observations to train and validate models that rely on remotely sensed data are critical, particularly in cases where resolution limits visual detection by the human eye or when overhanging vegetation obscures the channel.

Aside from modeling efforts, at a local scale, land managers can directly use these data collected by different agencies, or modeled output from these data, to inform their own decisions about water allocation permitting, conservation planning, or recreational use (Table 3). Often local organizations that could directly use these data do not have the resources for their own data collection efforts, underscoring the importance of public data accessibility.

## 6. Challenges with Compiling Water Presence Observations

Surface water presence (static or flowing) observations are relatively simple data to collect given that they are typically discrete (single observation at a point location) and generally categorical. As such, databases would presumably have relatively minimal metadata requirements that are limited to a data point identifier, date, categorical streamflow presence observation (flow, no flow, standing water), and geographical coordinates of latitude and longitude. However, consistency, geographic accuracy, accommodation of different types of data that include both discrete and time series data structures, and sampling error are legitimate challenges that must be considered in an effort toward an integrated database.

### 6.1. Consistency of Terminology and Methods across Sampling Programs

Linguistic uncertainty related to inconsistent and sometimes imprecise use of terms is a major source of confusion for understanding flow permanence [6]. A common vocabulary is not currently used to characterize presence/absence of streamflow across different field surveys. For example, terms guiding USGS mapping of blue line designations for streams use hydrographic categories (intermittent, perennial); other protocols use flowing/not flowing [35], surface water/no surface water [73], or other surface water presence surveys that can include the description of longitudinal connectivity of surface flow or wetted width at a cross section [74,75]. Even recently developed applications of CrowdWater, Stream Tracker, and FLOwPER field surveys have different categories of surface water presence conditions that do not necessarily align (Table 2). Collapsing these varying surface water categories into two basic categories of surface water presence or absence is straightforward; however, intermediate classes that describe spatially discontinuous flow, standing water, or unconnected pooled water can be challenging to align given the disparity in scales of observation among these three applications. For example, a “no flow” observation in Stream Tracker reflects the conditions immediately upstream and downstream of where a stream crosses a trail or road. This same condition may be considered “discontinuous flow” in FLOwPER if surface water is present over the larger observation area of 10 m. In this case, an observation that would be collapsed to dry from Stream Tracker would have been considered as a wet observation if observed in FLOwPER. These features of spatial intermittency are challenging to document, but they have important consequences for aquatic organisms and model development. Consequently, integrating several categories of surface water presence conditions will require additional interpretation with clear definitions and identified logic workflow to allow for flexibility to data end users [6].

### 6.2. Accuracy of Geographic Information and Association with a Hydrologic Network

Accurate spatial referencing of field observations on flow permanence is a critical issue that can be easily overlooked. Fundamentally, a surface water-presence observation is a spatially referenced point or line. Typically, these observations are referenced with Global Positioning System (GPS)-enabled mobile devices that record the latitude and longitude of the observation location. Accuracy of these point locations can vary substantially depending on device hardware, software, land cover, topography, satellite availability, and weather conditions, leading to varying degrees of uncertainty about the observation location.

**Figure 5.**
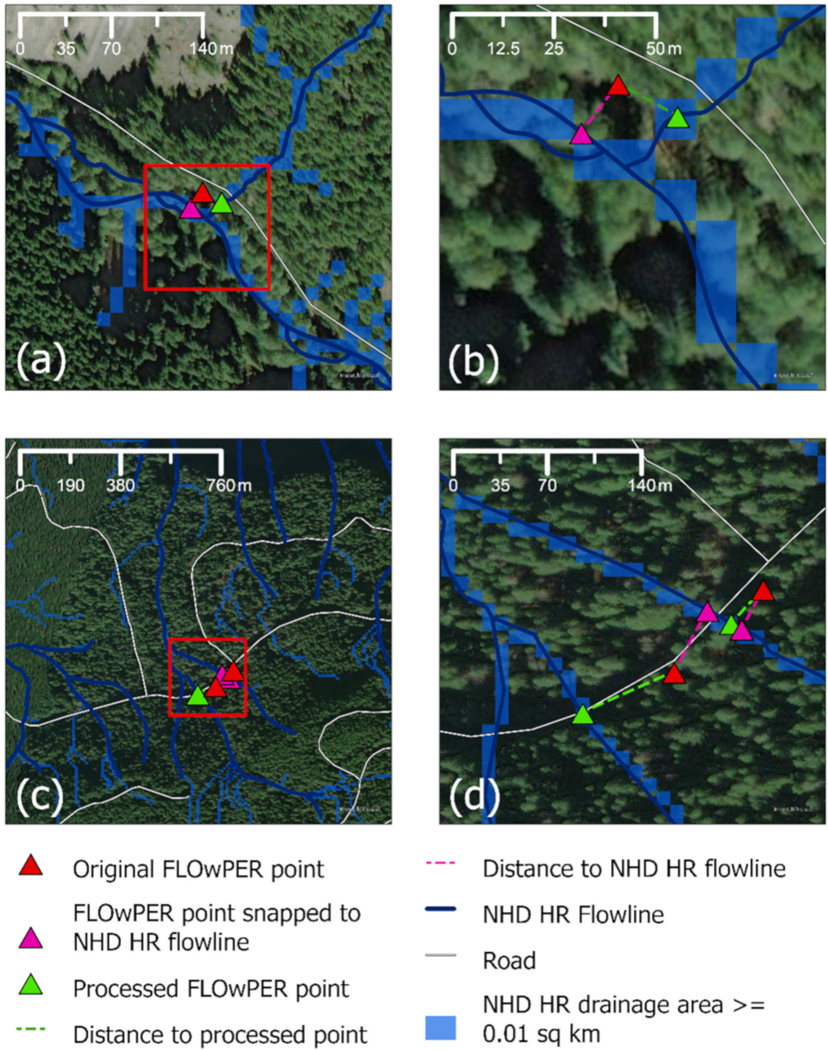
Examples of the challenge of assigning field observation to a stream network, in this case, the NHD High Resolution flowline and NHD High Resolution flow accumulation stream grid. Red triangles are original points, pink triangles are the point snapped to the closest NHD High Resolution flowline, and green triangles are the final processed locations on the NHD High Resolution flowlines and flow accumulation derived stream grid network. (**a**) An observation collected near a valley bottom is near the mainstem and a tributary that intersects the road. (**b**) Inset of (**a**). The original point is closer to the mainstem NHD flowline, though the snapping distance to the tributary is nearly equal. Information interpreted from the FLOwPER field form indicates that the observation should be assigned to the tributary. (**c**) Two observations collected in the headwaters along a road. (**d**) Inset of (**c**). The two original points are both closest to the flowline to the east. However, information interpreted from the FLOwPER field form and interpretation of the map indicate that the western observation should be assigned to the western tributary.

For further analysis of surface water presence observations, it is helpful to connect these observations to spatial datasets that represent stream networks. In the U.S., the most commonly used layers come from the NHD, which in most locations is available in medium (30 m, 1:100,000) and high (10 m, 1:24,000) resolution. Stream networks may also be derived from gridded topographic data; these are sensitive to the resolution of the data, the accuracy of the vertical dimension, the algorithm used to determine flow direction, and the thresholds selected to determine whether grid cells are part of the stream network [76,77]. For larger perennial streams, all of these data sources may be relatively consistent in stream location, but locations of small headwater streams vary more among data sources. Consequently, associating an observation point (GPS location) in the field with a stream flowline or stream grid cell often requires moving (or “snapping”) the location of the point. This process can be automated by identifying the nearest stream within some specified distance of the observation point. However, in many headwater locations, identifying the appropriate flowline or stream grid cell can be difficult, especially with an automated approach, if multiple potential stream features may be relatively close to the observation location (Figure 5). In many cases, end users processing the data must make judgement calls and manually assign observation points to a stream feature. Accurately capturing the geographic information of a field observation, therefore, is fundamental to facilitate post processing efforts that attribute surface water presence observations to a stream network. In addition, the high and medium resolutions of the NHD, both of which are widely used, are derived from different sources and resolutions, resulting in stream networks that do not necessarily align [24]. Flexibility to use both stream network resolutions while retaining the observation locality information will facilitate optimal use of the data.

### 6.3. Timing and Temporal Resolution

Most surface water presence observations are typically one-time, at-a-point observations, but some observations are repeated monthly or annually at a given location. In addition, other data types that include time series data of electrical resistivity sensors [40,78], thermistors [79,80], or multi-sensor systems [81] have been used as surrogates for surface water presence. These data series have a fundamentally different data structure relative to the discrete, one-time observation, and they have different metadata requirements to appropriately document the details including accuracy, precision, and timesteps of the measuring instrument as well as field installation details [82]. Both temporally intermittent and time series observations would be useful components of a streamflow permanence database as the two data types are complementary. The snapshot observations of streamflow presence/absence are more likely to cover a large number of streams spatially, and the time series observations can help infer flow conditions during times without snapshot observations.

### 6.4. Observation and Sampling Errors

For visual field observations that are discontinuous, the timing of field observations will affect the proportion of surface water presence/absence observations. Surveys timed only during low flow seasons are more likely to have a high proportion of absence observations than surveys conducted during high flow seasons or over multiple seasons. The intended temporal resolution to characterize the streamflow regime will determine the temporal distribution of observations needed. For example, observations timed only during low flow seasons may be useful in distinguishing perennial from non-perennial streams, but are not as useful for distinguishing ephemeral from intermittent streams.

Similarly, inconsistencies among observers using the same protocol or observations across different protocols introduce errors that must be considered. Sampling methods and determination of surface water condition can be subjective and therefore inconsistent across data collectors, even using the same vocabulary. For example, one observer might consider a flowing stream to have a “trickle” of flow, whereas another might classify the same stream as “flowing.” Finally, a stream may look “wet” to one observer because of recent rainfall that has directly wetted the streambed, whereas another observer might consider the same stream “dry” if they see no physical indicators that water had recently flowed through the channel beyond the presence of wet sediments. Although statistical approaches exist to address observation and sampling error (e.g., [83]), these are important considerations for end users of these data.

Finally, end users of these data will need to consider bias in sampling timing and intensity, acknowledging the bias in incidental streamflow permanence data mined from other stream surveys as well as opportunistic data collection using crowdsourced mobile apps. For example, incidental streamflow permanence data can be highly useful; however, these datasets were not collected with the goal of documenting streamflow status, so they may not be representative of the true variability and frequency of no flow conditions across these landscapes.

## 7. Next Steps

There are several examples of national-scale repositories that serve as starting points for an integrated database of surface water presence observations. As already mentioned, USGS NWIS (waterdata.usgs.gov/nwis (accessed on 15 February 2021)) is a publicly accessible database of streamflow, groundwater levels, water quality, sediment, and other variables. All published data in NWIS have undergone the established QAQC protocol, and the database structure generally facilitates aggregation or integration between the different datasets. Similarly, Environment and Climate Change Canada maintains a National Water Data Archive, HYDAT (https://www.canada.ca/en/environment-climate-change/services/water-overview/quantity/monitoring/survey/data-products-services/national-archive-hydat.html (accessed on 4 June 2021)), which can be downloaded as a stand-alone database. The Consortium of Universities for the Advancement of Hydrologic Science (CUAHSI) maintains Hydroshare (hydroshare.org (accessed on 4 June 2021)) to publish hydrologic datasets, which can be extensive. Hydroshare does not have specific metadata requirements. With over 120 streamflow data packages with the term “streamflow”, the Environmental Data Initiative (https://environmentaldatainitiative.org/ (accessed on 4 June 2021)) has become the common repository for some USFS Experimental Forests and Long Term Ecological Research (LTER) sites, many of which include headwater streams. Water quality data in the U.S. are available through the National Water Quality Monitoring Council (https://acwi.gov/monitoring/ (accessed on 4 June 2021)), which allows data to be queried based on several factors including station identifier and watershed identifier. Another example of an expansive water database is the Western States Water Council’s Water Data Exchange (https://www.wade.westernstateswater.org (accessed on 4 June 2021)), which provides and harmonizes water use and availability data for the western U.S. These repositories showcase a range of functionalities from URL-based query services (NWIS) to full database downloads (HYDAT). The creation of a new repository for surface water presence observations could use the best of these examples to maximize usability and maintainability.

The goals of a streamflow permanence database are to leverage the potentially vast amounts of data in existing data surveys, ensure accurate, high quality data collection going forward, and optimize utility to a broad end user audience through proper documentation in the metadata and flexible querying abilities. Based on our combined experience of both collecting streamflow permanence data and working with the different incidental streamflow permanence data for our individual applications, we propose a list of both critical and additional useful components to consider in development of a unified database (Table 4). The four critical components of a database of streamflow permanence observations are (1) date and time of observation; (2) geographical information associated with each observation; (3) surface water presence or flow status of the observation based on articulated definitions; and (4) comprehensive metadata to allow for reuse of the data (Table 4). Additional proposed components would increase the quality of the four critical components. Details included in each proposed component are examples and are not comprehensive.

To be most efficient, any new data collection should include high resolution temporal data with the reporting of date and time and the best available geographic location information (e.g., high-resolution GPS, sampling at points with highly resolved coordinates, and supporting map layers). Including known date and time is critical to place observations in the context of not only antecedent weather conditions at varying temporal scales (e.g., individual event, season, El Niño), but also land, water, and climate change. In addition, a fundamental component of each of the above listed stream water datasets is the known geographic location where the data were collected. Being able to place the point location in context of the surrounding landscape (topography, structures, road network), in addition to the stream network, provides additional confidence about the location accuracy when the observation is being recorded. Therefore, the more information the user can specify in terms of location and relationship to stream networks, the higher the quality of the surface water presence data. Similarly, more information provided to the observer in the form of NHD flowlines and topographic base layers can help users confirm the locations of their observations including actively attributing their observation to a specific flowline stream code. As accuracy in the hydrography improves and the NHD continues to be updated, accurate geographic location of field observations will facilitate efficient post processing. Source and accuracy in the geographical information of streamflow permanence observations, therefore, is included as an additional useful component of a database (Table 4). The surface water presence or flow status of observations will need to reconcile differences in terms and definitions across different field surveys and data collection approaches [6]. Observations that include a streamflow classification such as perennial, intermittent, and ephemeral may not be interchangeable or fully reconcilable with surface water presence/absence observations. Finally, proper documentation in the metadata that include definitions, description of methods, and explanation for all components of the data repository will facilitate use of the data.

A data repository should ideally have a way to reconcile existing data from incidental surveys (agency surveys), other data sources (e.g., Stream Tracker, FLOwPER), and data with different data structures (e.g., discrete one-time measurements versus time-series). Reporting information on data type and spatial and temporal scale of observation will support this goal, but the database framework will also have to accommodate different data structures. To facilitate flexibility in the use of the data repository, it should have end user features that include querying for repeat observations at a location and querying for observations within a given spatial area (e.g., a given flow line or stream reach). This will facilitate data use across a range of spatial and temporal scales specific to the end user without burdening the users with cumbersome post processing. Finally, while not a primary focus of the streamflow permanence mobile apps, presence/absence of a stream channel could be recorded as part of the field observation and serve as an ancillary data source to improve hydrography. As a result, the surface water feature of each observation is included as a useful component.

Given the urgency of the data needs, the building momentum for the collection of simplified hydrological data, and advances in database management, a national-scale repository of surface water presence observations is timely and attainable. This effort will require stakeholder buy-in and on-going involvement as it will require opening access of these sometimes-non-public datasets as well as metadata documentation of field protocols and any quality control measures associated with the data. However, establishing this database could lead to accelerating advances in research examining the drivers of streamflow permanence, which is critical information for improving streamflow permanence mapping nation-wide.

## Figures and Tables

**Table 1.** Current definitions of hydrographic classifications for Navigable Water Protection Rule compared to current and historical definitions of USGS hydrographic categories for NHD flowlines and area features classified as stream, river, or wash based on patterns of streamflow permanence.

Agency	Hydrographic Classification (USEPA *) or Category (USGS)	Definition

	Perennial	Surface water flowing continuously year-round.
	
USEPA and USACE ** definitions for streams, rivers, lakes [9]	Intermittent	Surface water flowing continuously during certain times of the year and more than in direct response to precipitation (e.g., seasonally when the groundwater table is elevated or when snowpack melts).
	
	Ephemeral	Surface water flowing or pooling only in direct response to precipitation (e.g., rain or snow fall).

	Perennial	Contains water throughout the year, except for infrequent periods of severe drought.
	
USGS NHD Flowline Feature and Area Feature: Stream or River [34]	Intermittent	Contains water for only part of the year, but more than just after rainstorms and at snowmelt.
	
	Ephemeral	Contains water only during or after a local rainstorm or heavy snowmelt.

USGS NHD Area Feature: Wash [34]	NA	The usually dry portion of a stream bed that contains water only during or after a local rainstorm or heavy snowmelt. May be a named feature.

**Historical USGS definitions for streams and rivers**		

	Perennial	Flows throughout the year.
	
Topographic instructions of Geological Survey, 1928 [35]	Intermittent	Dry for at least three months or longer.
	
	Ephemeral	None

	Perennial	Contains water more than 6 months of the year.
	
Topographic instructions of the USGS, 1955 [36]	Intermittent	Dry at least 6 months of the year.
	
	Ephemeral	None

USGS 2015 NHD Newsletter [37]	Ephemeral	Informally identified in some western U.S. states as intermittent streams mapped but unnamed in the NHD.

* USEPA: U.S. Environmental Protection Agency;

** USACE: U.S. Army Corps of Engineers.

**Table 2.** Examples of national and regional field surveys that include incidental streamflow permanence data and dedicated streamflow permanence data collection through community science and mobile applications. National and regional surveys include groups, example datasets, data collection purposes, and additional applications. Most states, agencies, and other jurisdictions have more localized monitoring programs that are not listed here. Data collection methods are all visual observations, with the exception of research settings where surrogate measures may be used as a proxy for flow conditions.

Host Agency/Organization	Program/Application Name	Purpose	Resolution	Categorization

**Incidental Observations Associated with Field Protocols**				

Bureau of Land Management (BLM)	Assessment Inventory Monitoring National Aquatic Monitoring Framework (Aquatic AIM)		Reach (minimum of 150 m or 20 m × bankfull width)	Streamflow Classifications: Intermittent /ephemeral distinction if flowing water in less than 5 transects
		
US Environmental Protection Agency (USEPA)	Environmental Monitoring and Assessment Program (EMAP), National Rivers and Assessment (NRSA)		40 × average wetted width; min 150 m, max 4 km	Reach has less than 50% water in the reach length, no data is collected. A dry cross section has a wetted width of 0 m, no macroinvertebrate samples taken.
		
Federal Interagency	PacFish/InFish Biological Opinion Monitoring (PIBO)	Monitoring for land, water and species management.	21–25 transects, 8–24 m apart	Flow, no flow, or other descriptive comments
		
National and State Parks	Inventory & Monitoring (I&M) Division		Unknown	Unknown
		
National/State Departments of Ecology/ Environmental Quality	Idaho DEQ Beneficial Use Reconnaissance Program (BURP)		Unknown	No ecological indicators are recorded on dry channels, “narrative” criteria apply to describe these conditions.
		
Federal Interagency	Aquatic and Riparian Effectiveness Monitoring Program (AREMP)		Reach, (160–480 m)	Note if a given transect is dry.

Universities and agencies	CUASHI HydroShare, local research watershed datasets	Research	Point, Reach, Network, Discrete, Timeseries	Direct measurements, surrogate measurements (e.g., temperature sensors), visual observations, etc.

**Dedicated Streamflow Permanence Observation through Community Science**				

Colorado State University	Stream Tracker		Point	3 categories: flow, standing water (pooled but not connected), no flow
		
University of Zurich	CrowdWater	Research	Point	5 categories: flowing, trickling water, standing water, isolated pools, damp/wet streambed, dry streambed

USGS/USFS R&D	FLOwPER	Research & Management	Reach (10 m)	3 categories: continuous flow, discontinuous flow, dry

**Table 3.** Applications of incidental streamflow class data beyond collecting agency.

**National-Regional-Subregional-State-Tribal Scale**

WOTUS determinations
Updating the NHD and facilitating concept of spatially and temporally dynamic NHD
Hydrologic modeling
Streamflow permanence modeling
Applying state and tribal water quality standards and aquatic life designations

**Local Scale**

Land manager decisions on water allocation (e.g., grazing rights, irrigation)
Identification of restoration, species repatriation, and land conservation projects
Environmental impact statements for mining and other development
Effect of withdrawals on surface water presence
Timber harvest riparian buffer widths based on streamflow status
Indirect or direct influence on extent and health of wetlands and wetland dwelling species
Recreational planning (anglers, boaters, drinking water sources for remote areas)

**Table 4.** Proposed components for a database for streamflow permanence observations.

Component	Description
**Critical data components**	
Date and time of observation	Includes time, day, month, and year. Observations without a date could receive a lower confidence value.
Geographical information	Coordinates and spatial data projection; associated with a streamline feature if known (e.g., NHD flowline).
Flow status	Surface water presence/absence, with sub-categories (e.g., continuous surface water/flow and discontinuous/standing water along a reach may collapse to flow, standing water, no surface water at a point).
	Streamflow classification: perennial, intermittent, ephemeral: may require several years of data to discern and may be problematic based on variation in classification definitions.
Comprehensive metadata	Clear definitions, method descriptions, quality control measures
**Additional useful components**	
Data type	Direct (visual observation [could include aircraft and unmanned aerial vehicle detection], sensor, and sensor type).
	Indirect/inferred (e.g., fish data collection may infer surface water presence, visual identification using imagery).
Scale of observation	Point, reach.
Temporal resolution	1-time observation, continuous time series including timestep and start-end dates.
Surface water feature definition	Natural channel, ditch, vegetated swale, sloped wetland, etc.
Observation confidence	A subjective assessment and may require a pre-determined rubric.
Data source	individual, report, online database, etc.
Accuracy of geographical information	Accuracy of GPS device on day of observation, source of geographical information and estimated accuracy from either direct or indirect observations.
Purpose of observation	May provide context and identify additional data sources (e.g., water quality, biological co-data collection).
